# Effect of restricted feeding and refeeding on the compensatory growth and serum metabolites of juvenile Siberian sturgeon (*Acipenser baerii*)

**DOI:** 10.3389/abp.2025.14761

**Published:** 2025-08-14

**Authors:** Bahram Falahatkar, Ali Razgardani Sharahi, Ahmad Nosrati Movafagh, Belal Moludinia

**Affiliations:** ^1^ Fisheries Department, Faculty of Natural Resources, University of Guilan, Sowmeh Sara, Guilan, Iran; ^2^ Department of Marine Sciences, The Caspian Sea Basin Research Center, University of Guilan, Rasht, Guilan, Iran; ^3^ Fisheries Department, Faculty of Animal Science and Fisheries, Sari University of Agricultural and Natural Science Resources, Sari, Iran

**Keywords:** catch-up growth, feed restriction, hematology, Siberian sturgeon, nutrition

## Abstract

During the rearing period, fish may be exposed to fasting due to low or high temperatures, transportation, handling, and other stressors, while they may catch-up the growth differently after supplying the feed. The aim of this study was to investigate the compensatory growth (CG) response of juvenile Siberian sturgeon *Acipenser baerii* after restricted feeding. In the first phase (60 days), triplicate groups of fish were subjected to feed restriction (25%, 50%, and 75% of the amount needed to reach satiation, respectively) or satiation feeding (control) and in the second phase all treatment groups were fed to satiation for an additional 60 days. Growth performance was measured during the two phases and blood samples were collected at the end of the second phase to measure blood biochemical indices. At the end of the first phase of the experiment, as expected, the control group showed the highest mean body weight, followed by the 75%, 50% and 25% satiation fed groups (*P* < 0.05). However, at the end of the second phase, the final body weights were not significantly different between the groups (*P* > 0.05), while some of the growth performance improved in the 25% satiation fed group (*P* < 0.05). At the end of the second phase, serum metabolites except for glucose and cholesterol were significantly different among treatment groups, with the highest levels in the control group. Feed restriction significantly lowered hematocrit, total protein and triglyceride levels especially in the 25% satiation fed group (*P* < 0.05). The results showed that juvenile Siberian sturgeon could tolerate feed restriction without any significant negative impacts on the majority of growth and metabolite indices. In conclusion, using an appropriate feeding regime helps to improve feed efficiency with no physiological impacts on Siberian sturgeon rearing.

## Introduction

Compensatory growth (CG) is a fast and natural biological response to prior conditions that happens when adequate refeeding follows restricted food availability or unfavorable environmental conditions (McCue, 2010; Won and Borski, 2013). This event may be due to hyperphagia (appetite acceleration), improved feed efficiency, and enhanced protein synthesis or energy reserve replacement (Urbinati et al., 2014; Xiao et al., 2013). Using a feeding strategy based on CG can help reduce feed costs, minimize waste load and reduce labor costs. Although several studies have been conducted on the CG of different fish species, the results have been inconsistent. For instance, full compensation (Tian and Qin, 2003; 2004; Xie et al., 2001), or partial compensation (Ashouri et al., 2020; Wang et al., 2000; Yarmohammadi et al., 2012) have been observed in different fish species. However, no compensation (Falahatkar, 2012) and overcompensation (Chatakondi and Yant, 2001; Hayward et al., 1997) have also been reported. Therefore further research is needed to understand CG efficiency in different species.

The growth and feed efficiency of fish species are two of the most critical economic parameters that influence the profitability of production. As feed constitutes approximately 50% of the total production cost in modern aquaculture (Rana et al., 2009); therefore, applying appropriate feeding strategies can increase profits and decrease waste. Restricted feeding followed by refeeding is expected to induce CG, allowing lost weight to be regained. This technique may be practically applicable in aquaculture and proper management of the feeding schedule may allow for the design of feeding regimes that improve growth rates, feed efficiency and production costs. Additionally, studying the physiological aspects of this process could provide a better understanding of compensation mechanisms and provide a basis for further studies. Previous studies have shown that CG could affect serum metabolites such as glucose, triglycerides, cholesterol and total protein levels, which are good indicators for monitoring CG occurrence (Falahatkar, 2012; Shirvan et al., 2019).

Sturgeons are important cultured species worldwide due to their meat and caviar. To successfully produce and adapt sturgeon to farm environments, more data on their nutritional requirements and feeding strategies are needed (Falahatkar, 2018). The Siberian sturgeon (*Acipenser baerii*) is an important chondrostean species for research on sturgeon physiology (Eslamloo et al., 2012; Rodriguez et al., 2002) due to its high production value, abundance, and early puberty age compared to most other sturgeon species. Previous studies on growth performance in Siberian sturgeon have mainly focused on feed macronutrients (Mohseni et al., 2007; Yun et al., 2014; Zhu et al., 2011). Moreover, the majority of studies on CG in sturgeons have focused on complete feed deprivation (Falahatkar, 2012; Liu et al., 2011; Morshedi et al., 2013; Florescu et al., 2019; Ashouri et al., 2020) and the CG response to restricted feeding prior to satiation feeding has rarely been studied. Throughout the rearing period, sturgeon may be exposed to fasting or restricted feeding because of low or high temperatures, transportation, handling, or any other stressors, while they may catch-up the growth differently after feed is supplied. Therefore, this study aimed to investigate the effects of restricted feeding at different levels and refeeding on CG and some serum metabolites in juvenile Siberian sturgeon.

## Materials and methods

### Animal ethics

The animal study was reviewed and approved by our university and according to the ethical guidelines outlined in the National Ethical Framework for Animal Research.

### Fish, culture system and experimental design

Siberian sturgeon were obtained from the Shahid Dr. Beheshti Sturgeon Restoration and Genetic Conservation Center (Guilan, Iran) and transported to the experimental facility at the wet lab in the Faculty of Natural Resources, University of Guilan (Sowmeh Sara, Guilan, Iran). The fish, with a mean weight of 66.80 ± 1.03 g, were acclimated for 2 weeks to the new culture conditions and fed until satiated. Four treatment groups with three replicates were used in this study. Twelve 500-L indoor fiberglass tanks were stocked with 8 fish in each tank. The system consisted of a flow-through system that was supplied with well water at a flow rate of 7.80 ± 1.66 L min^−1^ under a 12L:12D photoperiod. The experiment was conducted in a completely randomized design over two sixty-day phases (for a total of 120 days). In the first phase (restricted feeding), the fish were fed with the following feeding rations: 1) 100% satiation (control group), 2) 75% satiation, 3) 50% satiation, and 4) 25% satiation. These levels of feeding were selected based on the previous studies (Jafari et al., 2019; Shirvan et al., 2019). In the second phase, all fish were fed to 100% satiation. To maintain water quality, 25% of the water in each tank was siphoned out daily before the first feeding. Throughout the experimental period, water quality parameters such as temperature (18.28°C ± 0.13°C), dissolved oxygen (6.89 ± 0.19 mg L^−1^) and pH (7.39 ± 0.11) did not differ between treatment groups. These water quality parameters were within the optimal range for sturgeon (Chebanov and Galich, 2013). The fish were fed three times a day at 8:00 a.m., 1:00 p.m., and 6:00 p.m. with sinking commercial feed (Skretting, Verona, Italy) containing 48% crude protein and 22% crude fat. In the first phase, the satiation level (when the fish stopped consuming feed) was determined by the mean consumption of 3 control tanks and the other fish were fed with restricted rations accordingly. To determine this level, the fish were fed until they stopped consuming feed, and that amount was considered the level of satiation. Feeding for this group continued for up to 30 min, during which time the fish did not consume feed particles.

### Sample collection

At each 20-day interval, the fish were fasted for 24 h before weighing and were anesthetized with 300 mg L^−1^ clove powder extract to reduce stress. The individual body weight and total length of all fish were measured to the nearest 0.01 g and 1 mm, respectively. At the end of the second phase, after starving the fish for 24 h, three individuals from each tank were randomly captured and anesthetized. Two mL of blood were withdrawn from the caudal vein of each fish using a 2 mL non-heparinized syringe. One and a half mL of the collected blood was transferred to a non-heparinized tube for further serum metabolite analysis. The remaining blood was transferred to microhematocrit tubes to measure hematocrit values. Serum samples were obtained after centrifugation of clotted blood (3000 × *g*, 10 min, 4°C) and then stored at −80°C for further analyses.

Growth performance and feed efficiency were assessed in terms of weight gain (WG), body weight increase (BWI), condition factor (CF), specific growth rate (SGR), feed intake (FI), feed conversion ratio (FCR), protein efficiency ratio (PER), lipid efficiency ratio (LER) and survival rate (SR) using the following formulas (Falahatkar, 2012):

WG (g) = final body weight - initial body weight
  BWI (%) = 100 × (final weight - initial weight)/initial weight
  CF = 100 × (body weight/body length^3^)
  SGR (% day^-1^) = 100 × (Ln final body weight - Ln initial body weight)/days
  FI (g) = consumed feed/fish number
  FCR = feed intake/WG
  PER = WG/protein intake
  LER = WG/lipid intake
  SR (%) = 100 × (final number of fish/initial number of fish)

### Hematological and biochemical analyses

Hematocrit (Hct) was determined by centrifuging (Hettrich, Tuttlingen, Germany) whole blood in heparinized microhematocrit capillary tubes at 3500 × *g* for 10 min (Falahatkar, 2012). Serum glucose, total protein (TP), cholesterol (CHL) and triglyceride (TG) concentrations were determined using available commercial kits (Pars Azmun, Karaj, Iran) based on colorimetric reactions and a spectrophotometer (Unico, 2100-VIS, USA) at 546 nm (Bradford, 1976).

### Statistical analysis

All statistical analyses were conducted using SPSS software, version 16 (Chicago, IL, USA). The normality of the data and the homogeneity of the variances were checked using the Kolmogorov-Smirnov and Levene’s tests, respectively. Comparison between different groups was analyzed by One-way analysis of variance, and Tukey’s multiple range tests at *P* > 0.05 were used to determine significant differences between means when necessary. Values are expressed as mean ± standard error (SE). For the second phase, growth parameters were analyzed by analysis of covariance. Initial weight on day 60 was used as a covariate in the repeated-measures analysis of covariance (ANCOVA).

## Results

During the experimental period, no mortality was observed. After 60 days of feed restriction, growth and feed efficiency except for CF differed among all feeding groups (*P* < 0.05; Table 1). Final body weight, final length, WG, SGR, BWI and FI gradually decreased, with the control group obtaining the highest values, followed by the 75%, 50% and 25% satiation-fed groups (*P* < 0.05). The highest and lowest FCR values were observed in the control and 50% satiation groups, respectively (*P* < 0.05). The highest PER and LER were observed in the 50% satiation group (*P* < 0.05) and the lowest PER was observed in the control group, which had no significant difference with the 25% and 75% satiation groups (*P* > 0.05), however, the LER was significantly lower in the control than in the other groups (*P* < 0.05).

**TABLE 1:** Growth performance of Siberian sturgeon *Acipenser baerii* after 60 days of restricted feeding. Values are means ± SE (n = 3). Different superscripts in the same row indicate a significant difference (*P* < 0.05) between groups.

Parameters	Satiation rate (%)			
	25	50	75	100
Initial weight (g)	66.71 ± 1.04	66.80 ± 0.84	66.94 ± 0.98	66.78 ± 0.99
Final weight (g)	104.89 ± 0.61^w^	152.44 ± 1.01^x^	182.55 ± 6.32^y^	204.74 ± 11.43^z^
Initial length (cm)	27.02 ± 0.23	27.42 ± 0.25	27.48 ± 0.20	27.86 ± 0.29
Final length (cm)	32.83 ± 0.27^x^	36.46 ± 0.26^y^	38.99 ± 0.54^z^	39.66 ± 0.43^z^
WG (g)	38.19 ± 1.19^w^	85.64 ± 0.54^x^	115.61 ± 6.71^y^	137.96 ± 10.89^z^
CF	0.30 ± 0.01	0.31 ± 0.01	0.31 ± 0.00	0.33 ± 0.01
SGR (% day^−1^)	0.76 ± 0.07^x^	1.37 ± 0.09^y^	1.67 ± 0.11^z^	1.87 ± 0.13^z^
BWI (%)	57.27 ± 2.30^w^	128.20 ± 0.36^x^	172.83 ± 11.09^y^	206.36 ± 14.60^z^
FI (g fish^−1^)	34.83 ± 0.21^w^	69.73 ± 0.35^x^	104.81 ± 0.84^y^	139.32 ± 0.12^z^
FCR	0.91 ± 0.03^w^	0.81 ± 0.01^x^	0.91 ± 0.06^y^	1.02 ± 0.08^z^
PER	5.61 ± 0.17^y^	6.29 ± 0.04^z^	5.65 ± 0.33^y^	5.08 ± 0.42^y^
LER	1.64 ± 0.05^y^	1.84 ± 0.01^z^	1.66 ± 0.09^y^	1.49 ± 0.11^x^
SR (%)	100	100	100	100

WG, weight gain; CF, condition factor; SGR, specific growth rate; BWI, body weight increase; FI, feed intake; FCR, feed conversion ratio; PER, protein efficiency ratio; LER, lipid efficiency ratio; SR, survival rate.

In the second phase after 60 days of feeding to satiation, the highest CF, SGR and BWI were found in 25%-fed fish in the previous feeding scheme (*P* < 0.05, Table 2), while no differences were observed for the other parameters (*P* > 0.05). Final length was significantly higher in the control than in the 25%- and 50%-fed groups (*P* < 0.05).

**TABLE 2:** Growth performance of Siberian sturgeon *Acipenser baerii* after 60 days of feed restriction followed by 60 days of normal refeeding. Values are means ± SE (n = 3). Different superscripts in the same row indicate a significant difference (*P* < 0.05) between groups.

Parameters	Satiation rate (%)			
	25	50	75	100
Initial weight (g)	104.89 ± 0.61^w^	152.44 ± 1.01^x^	182.55 ± 6.32^y^	204.74 ± 11.43^z^
Final weight (g)	262.53 ± 9.78	285.82 ± 16.89	311.83 ± 12.35	314.23 ± 9.59
Initial length (cm)	32.83 ± 0.27^x^	36.46 ± 0.26^y^	38.99 ± 0.54^z^	39.66 ± 0.43^z^
Final length (cm)	41.29 ± 0.51^y^	43.09 ± 0.77^y^	45.06 ± 0.72^yz^	48.99 ± 0.42^z^
WG (g)	157.64 ± 3.99	133.38 ± 6.62	129.31 ± 6.38	109.49 ± 7.48
CF	0.37 ± 0.01^z^	0.35 ± 0.01^yz^	0.33 ± 0.01^y^	0.33 ± 0.01^y^
SGR (% day^-1^)	1.53 ± 0.06^z^	1.04 ± 0.31^y^	0.88 ± 0.11^y^	0.72 ± 0.07^y^
BWI (%)	150.25 ± 2.95^z^	87.64 ± 2.03^z^	70.11 ± 2.65^z^	53.41 ± 0.71^y^
FI (g fish^-1^)	130.80 ± 8.03	144.11 ± 7.13	156.33 ± 7.33	130.15 ± 7.58
FCR	0.82 ± 0.05	1.09 ± 0.07	1.21 ± 0.05	1.22 ± 0.24
PER	2.51 ± 0.02	1.92 ± 0.13	1.72 ± 0.08	1.75 ± 0.33
LER	5.48 ± 0.04	4.20 ± 0.28	3.76 ± 0.17	3.81 ± 0.71
SR (%)	100	100	100	100

WG, weight gain; CF, condition factor; SGR, specific growth rate; BWI, body weight increase; FI, feed intake; FCR, feed conversion ratio; PER, protein efficiency ratio; LER, lipid efficiency ratio; SR, survival rate.

**FIGURE 1:**
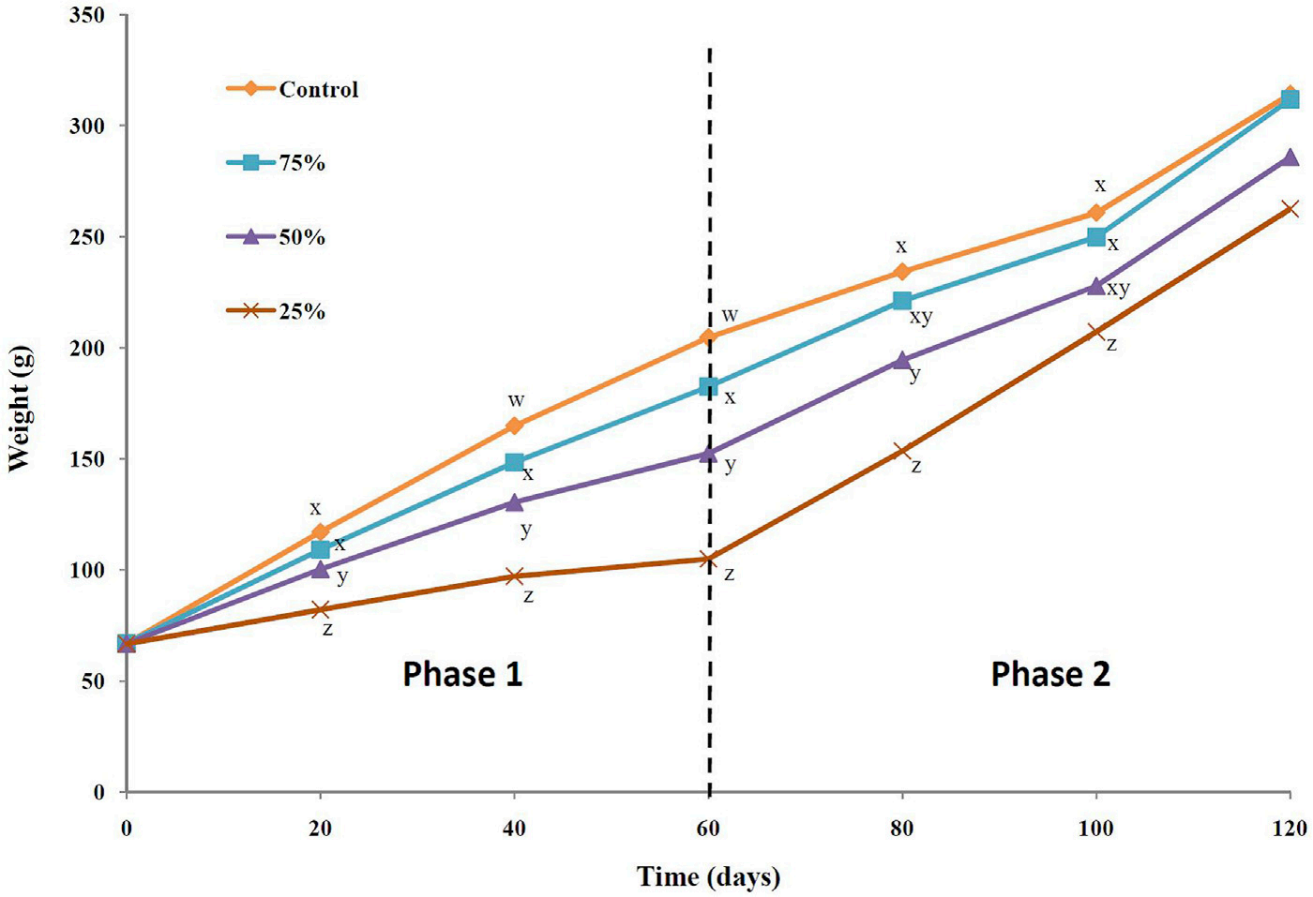
Biweekly changes in body weight of Siberian sturgeon *Acipenser baerii* during feed restriction (0–60 days: phase 1) and normal refeeding (61–120 days: phase 2) experiment in relation to four feeding strategies. Values are the mean ± standard error (SE) of triplicate groups. Different superscripts on each day represent statistical difference among the treatment groups (*P* < 0.05).

Biweekly changes in the body weight of Siberian sturgeon during the 120-day experiment are shown in Figure 1. The weight of the fish was affected by the feeding/fasting strategies, with the highest and lowest body weights observed in the control and 25% satiation groups, respectively, and CG observed after normal refeeding.

As shown in Table 3, at the end of the second phase, blood parameters except glucose and CHL showed significant differences among treatment groups (*P* < 0.05). The lowest Hct, TP and TG were observed in the 25%-fed group (*P* < 0.05).

**TABLE 3:** Hematocrit and serum metabolites of the Siberian sturgeon *Acipenser baerii* after 60 days of feed restriction followed by 60 days of normal refeeding. Values are means ± SE (n = 3 from each replicate). Different superscripts in the same row indicate a significant difference (*P* < 0.05) between groups.

Parameters	Satiation rate (%)			
	25	50	75	100
Hematocrit (%)	26.93 ± 0.98^y^	31.74 ± 1.12^z^	30.53 ± 0.92^z^	31.35 ± 0.38^z^
Total protein (g dL^-1^)	2.50 ± 0.63^y^	2.77 ± 0.61^yz^	2.97 ± 0.49^z^	3.03 ± 0.91^z^
Glucose (mg dL^-1^)	45.64 ± 1.19	45.53 ± 2.48	44.79 ± 1.99	45.44 ± 2.14
Cholesterol (mg dL^-1^)	89.31 ± 10.45	95.81 ± 7.05	103.24 ± 6.17	113.76 ± 8.02
Triglyceride (mg dL^-1^)	184.10 ± 19.76^x^	252.05 ± 11.45^y^	331.50 ± 10.17^z^	367.11 ± 18.14^z^

## Discussion

### Growth performance

In this study, we examined the effects of feed restriction, followed by satiated feeding on the CG and physiological responses of Siberian sturgeon. The results clearly demonstrate that varying degrees of feed restriction significantly decreased most growth parameters compared to the control group. It is evident that feed restriction leads to rather slower weight gains and there is a linear correlation between growth rate and feed intake (Peres and Oliva-Teles, 2005). Similar reductions in growth have been recorded in previous studies (Liu et al., 2011; Srijila et al., 2014). In a similar study on juvenile great sturgeon (*Huso huso*), during winter feeding at 10°C, found that the highest growth performance occurred in fish fed 1% of their body weight/day and in fish fed to satiation; however, negative growth rates were observed in fish fed 0.2% body weight per day (Falahatkar et al., 2012). Although the best growth rate of Siberian sturgeon was observed in the group fed to 100% satiation, the best FCR, PER and LER were observed when fish were fed to 50% satiation. In feed-restricted treatment groups, a portion of the given feed is likely to be digested and absorbed more completely with longer retention times, leading to an increase in nutrient conversion (Mohseni et al., 2006), and optimizing metabolic partitioning toward growth rather than maintenance or waste.

By the end of the second phase (following refeeding), CG had been fully achieved across all restricted groups, eliminating significant differences in final weight compared to the control group. CG refers to the ability of animals to grow fast over a time period when fed varying amounts of food after experiencing a reduced growth period. CG can be induced by restricted food availability or other unfavorable environmental conditions (Wang et al., 2009). In the present study, the 100% satiation group had a higher final weight than the feed-restricted groups, but no significant difference was found among treatment groups, so that full CG was observed in the restricted groups, while the length of fish was smaller than that in the restricted groups. However, Yarmohammadi et al. (2012) observed complete CG in Persian sturgeon (*Acipenser persicus*) that were only fasted for 1 week and then refed for several more weeks. Furthermore, Siberian sturgeon that were fasted for 1, 2 or 3 weeks did not attain the final weight of the control group after 4 weeks of the refeeding phase (Ashouri et al., 2020). Sirjila et al. (2014) observed overcompensation in Rohu *Labeo rohita* after 6 weeks of feed restriction and subsequent 6 weeks of restarting the feeding in the 75% restriction group. These different responses may be attributed to the growth compensation ability of different species, along with life stage, feed type/quality, the duration and intensity of feed deprivation and refeeding schedule, in addition to the rearing temperature (Mohanta et al., 2016).

In the present study, restricted feeding had an adverse effect on SGR, but when food was provided to the restricted fish, the SGR improved. This improvement was only observed in the 25%-fed treatment group, in which the SGR increased from 0.76% to 1.53% day^−1^ from the first to the second phase. In contrast, the SGR declined substantially (1.37–1.04, 1.67 to 0.88, and 1.87 to 0.72% day^-1^) between phases in the 50%-, 75%-, and 100%-fed treatment groups. Previous studies have indicated that the degree of CG is correlated with the severity of prior feed restriction, with the most noticeable differences in growth rates seen in fish that had the poorest growth during the period of feed restriction (Jobling and Johansen, 1999; Sæther and Jobling, 1999; Tian and Qin, 2003).

In this study, the FCR and LER improved after the second phase in the restricted-fed groups, which could be due to better digestibility and nutrient consumption. The FCR only improved between phases in the 25%-fed treatment group (from 0.91 to 0.82), whereas the other treatment groups showed a poorer FCR in the second phase. Similarly, the PER declined in all treatment groups between phases. The LER was the only factor that improved from phase 1 to phase 2. A better FCR in fish experiencing CG was reported in similar studies (Ribeiro and Suzuki, 2010; Falahatkar, 2012; Mohanty, 2015; Mohanta et al., 2016). Future studies should consider the mechanism by which CG affects fish. Decreased metabolic costs, reduced basal metabolism, increased feed conversion efficiency, efficient use of dietary protein, hyperphagia and adjustments of the endocrine system are the main reasons behind CG (Davis and Gaylord, 2011; Känkänen and Pirhonen, 2009; Sevgili et al., 2012; Sevgili et al., 2013). Dar et al. (2018) demonstrated that increased ghrelin levels and decreased leptin levels cause hyperphagia at the beginning of restarting the feeding, which further activates CG in Rohu. After feeding was restarted normally, hyperphagia was observed in the medium feed-restricted groups (25% and 50% satiation); however, feed intake did not differ significantly from that of the control group (100% satiation). This finding is consistent with the results of Sæther and Jobling (1999) who suggested that restricted feeding (0.25%, 0.38% and 1% of body weight day^-1^) in turbot (*Scophthalmus maximus*) caused slight hyperphagia (although not significantly) and in turn displayed CG after changing from restricted to excess feeding, with CG being most marked among fish that had been subjected to the most severe feed restriction. Siberian sturgeon exhibit a robust capacity for growth compensation following restriction, driven by physiological adaptations such as hyperphagia and metabolic efficiency. Further experiments should consider the effect of refeeding after starvation on ghrelin and leptin levels, along with that on key digestive enzymes (e.g., proteases, lipases).

### Physiological performance

In the current study, the levels of some serum metabolites in juvenile Siberian sturgeon were significantly altered among the treatment groups. After normal refeeding, the lowest Hct was observed in the 25% satiation group while there were no significant differences among the other treatment groups. Previous studies have reported different responses regarding the effects of starvation on hematological parameters, with starvation causing a decrease (Falahatkar, 2012; Gillis and Ballantyne, 1996; Lim and Klesius, 2003; Rios et al., 2005), an increase in Hct (Shoemaker et al., 2003) or sometimes no change (Caruso et al., 2011) reported. Typically, changes in body water content (McCue, 2010) and erythropoietic depression (Weinberg et al., 1973) are the main reasons for changes in Hct during starvation. Therefore, the significant reduction in Hct in the 25% satiation fed group can be attributed to the severity of the condition, resulting in malnutrition, and the fish’s inability to maintain body water content through appropriate osmoregulation or to replace lost blood cells.

The absence of nutrients is compensated for through the catabolism of energy resources (Navarro and Gutiérrez, 1995); however, prolonged feed restriction could deplete the body’s energy resources, resulting in decreased serum glucose, TG and CHL levels (Pérez-Jiménez et al., 2007). In the majority of species, after a short period of restarting feeding the metabolite content returns to pre-starvation levels (Furné et al., 2012; Morshedi et al., 2013). In the present study, after the refeeding period the lowest and highest concentrations of TG and CHL were observed in the 25% and 100% satiation fed groups, respectively. Previous studies have shown that during feed restriction the utilization of fatty acids for energy requirements is enhanced (Hornick et al., 2000) and lipids are the preferred nutrient over protein in terms of utilization; in addition sturgeon can be able to preserve muscle proteins better than lipids during feed restriction (Falahatkar et al., 2012). Pérez-Jiménez et al. (2007) reported that plasma TG increased in feed-deprived European seabass (*Dicentrarchus labrax*) and decreased after feeding. Furthermore, Ashouri et al. (2020) reported that after one to three weeks of fasting, the plasma concentrations of TG decreased in Siberian sturgeon, but the levels of total CHL increased. Free fatty acids, which are created through lipolysis, may be used for CHL synthesis, leading to hypercholesterolemia during the fasting phase (Thampy, 1995). In the present study, the reduction of both TG and CHL together in the feed-restricted groups could be attributed to the complete depletion of lipid reserves and the subsequent effects on serum metabolites due to the long feeding restriction time. Reduced TP levels together with a reduction in lipid resources indicate that amino acids were used as energy sources (Echevarría et al., 1997). Hung et al. (1997) found a significant decrease in TG levels in white sturgeon (*Acipenser transmuntanus*) after 10 weeks of fasting. The lowest level of TG was observed in fully starved great sturgeon (Falahatkar, 2012). Moreover, the serum plasma CHL concentration of Adriatic sturgeon (*Acipenser naccarii*) decreased after 40 days of starvation (Furné et al., 2008). Based on our findings, it appears that even after normal refeeding more time is needed to attain normal serum metabolites, likely due to the prolonged lower feed rates initially.

In the present study, there was a decrease in serum TP content especially in the 25% satiation-fed group compared with the 75%-fed and control groups. If the deficiency in feed persists, then the replacement of lipid by protein as the main metabolic fuel can increase (Hevrøy et al., 2011), so one of the main characteristics of CG is protein synthesis (Ali et al., 2003). Therefore, the reason for reduced TP in the severely restricted treatment group may be due to protein depletion during feed restriction. However, no significant difference in serum TP content was observed in starved groups after CG in olive flounder (*Paralichthys olivaceus*) fed diets with different nutrients (high protein, high carbohydrate, high lipid or intermediate) followed by 2 weeks of starvation (Cho, 2012). Our study confirms the results of Ashouri et al. (2020), who found that the plasma TP in severely fasted Siberian sturgeon was significantly lower than that of the control group after 4 weeks of refeeding. The difference in results may be attributed to the experimental protocol, rearing conditions and the severity of CG in the various treatment groups. The lack of TP recovery even after refeeding suggests persistent protein deficits or delayed synthetic capacity in severely restricted fish.

Despite changes of TG and TP, the serum glucose content was not affected by different feeding strategies, likely due to the ability of Siberian sturgeon to maintain or rapidly recover serum glucose after refeeding. Maintaining blood glucose concentration during starvation points to an active gluconeogenic process in the liver (Gillis and Ballantyne, 1996). The ability to maintain plasma glucose levels during fasting has been reported in Persian sturgeon (Yarmohammadi et al., 2012) and Lake sturgeon (*Acipenser fulvescens*) (Gillis and Ballantyne, 1996). However, some studies on sturgeons have shown a significant decrease in plasma glucose levels during the starvation period (Ashouri et al., 2020; Hung et al., 1997). Moreover, the plasma glucose content of small Siberian Sturgeon (with 40 g weight) significantly decreased after one to 3 weeks of starvation and did not recover after 4 weeks of refeeding. Plasma glucose levels can vary based on fish size in Siberian sturgeon subjected to feed restriction and deprivation; therefore, smaller fish may not be able to maintain serum glucose at normal levels (Jafari et al., 2018). In the present study, we used larger fish with restricted feeding instead of complete feed deprivation, which may explain the different results. Further studies should focus on the physiological alterations and different mechanisms that fish can use to tolerate starvation and supply energy.

### Conclusion

In conclusion, the results showed that refeeding of previously feed-restricted Siberian sturgeon induced CG and the response was not related to the severity of the feed restriction. Additionally, after normal refeeding complete CG can be achieved in moderately feed-restricted treatment groups. The degree of the response was related to the severity of feed restriction: for example, the 25%-fed group showed the greatest improvement in SGR and FCR between phases compared to the other treatment groups, which indicates the best utilization of food and recovery. Complete CG was achieved in all restricted groups (25%, 50%, 75% satiation) after refeeding, which eliminated the difference in final weight with controls. Refeeding after restricted feeding could not recover Hct or serum metabolites such as TG and TP after 60 days. Implementing such a severe restriction scheme commercially requires a careful risk-benefit analysis. According to growth performance and economic considerations, using a 50% feed restriction and refeeding strategy helps to improve feed efficiency in Siberian sturgeon rearing. Reducing the feeding rate and taking advantage of the CG response can be considered a way to increase productivity and profits in sturgeon culture especially during unfavorable rearing conditions.

## Data Availability

The original contributions presented in the study are included in the article/supplementary material, further inquiries can be directed to the corresponding author.
